# SAA1 and metabolomic signatures predict hyperprogression with immunotherapy in pan cancers

**DOI:** 10.1002/ctm2.1624

**Published:** 2024-03-11

**Authors:** Xiaoqing Wang, Longshan Zhang, Liwei Liao, Nan Li, Tingxi Tang, Jianda Sun, Zhenhua Zhou, Yang Liu, Jihong Huang, Yingqiao Wang, Zekai Chen, Hanbin Zhang, Ting Xiao, Yunming Tian, Xiuting Zheng, Yi Yuan, Linlin Xiao, Laiyu Liu, Jian Guan

**Affiliations:** ^1^ Department of Radiation Oncology Nanfang Hospital, Southern Medical University Guangzhou China; ^2^ Department of Radiation Oncology Meizhou People's Hospital Meizhou China; ^3^ Department of Respiratory and Critical Care Medicine Chronic Airways Diseases Laboratory Nanfang Hospital, Southern Medical University Guangzhou China; ^4^ Sun Yat‐sen University Cancer Center, State Key Laboratory of Oncology in South China, Collaborative Innovation Center for Cancer Medicine, Guangdong Key Laboratory of Nasopharyngeal Carcinoma Diagnosis and Therapy Guangzhou China; ^5^ Guangdong Province Key Laboratory of Molecular Tumor Pathology Guangzhou China

Dear Editor,

Hyperprogression disease (HPD) has been identified as a special form of progression correlated to immune checkpoint inhibitor (ICI) treatment and is featured by a sudden and dramatic acceleration of tumour progression following ICI treatment, dramatically reducing survival time.^1^, ^2^ There are limited strategies to address this clinical dilemma.^3^ Thus, the implementation of valid predictive biomarkers is urgently needed. Unfortunately, there are few available biomarkers for identifying HPD.

In this study, we met with an HPD patient. Briefly, the patient was diagnosed with metastatic nasopharyngeal carcinoma and treated with camrelizumab. Unfortunately, an unexpected and dramatic acceleration of tumour progression occurred after immunotherapy (Figure S1A,B), thus the patient was diagnosed with HPD. Importantly, we prospectively collected tumour tissue and dynamic blood samples (Table S1) throughout the entire treatment process, which provided support for exploring the potential mechanisms and biomarkers of HPD. Moreover, two patients without HPD with matched baselines were enrolled for comparative analysis. We performed a multidimensional analysis based on integrated tumour tissues and plasma data (Figure 1A). Genetic sequencing indicated that the gene mutations in plasma were highly concordant with those in tumour tissues (Table S2), suggesting that plasma ctDNA may be an effective alternative to testing for patients whose tissue biopsies are not obtained. Mutation assessment indicated that the neurofibromin 1 gene (NF1), NFKB inhibitor alpha gene (NFKBIA), and tumour protein p53 gene (TP53) mutations might be oncogenic mutations in HPD (Figure 1B).

**FIGURE 1:**
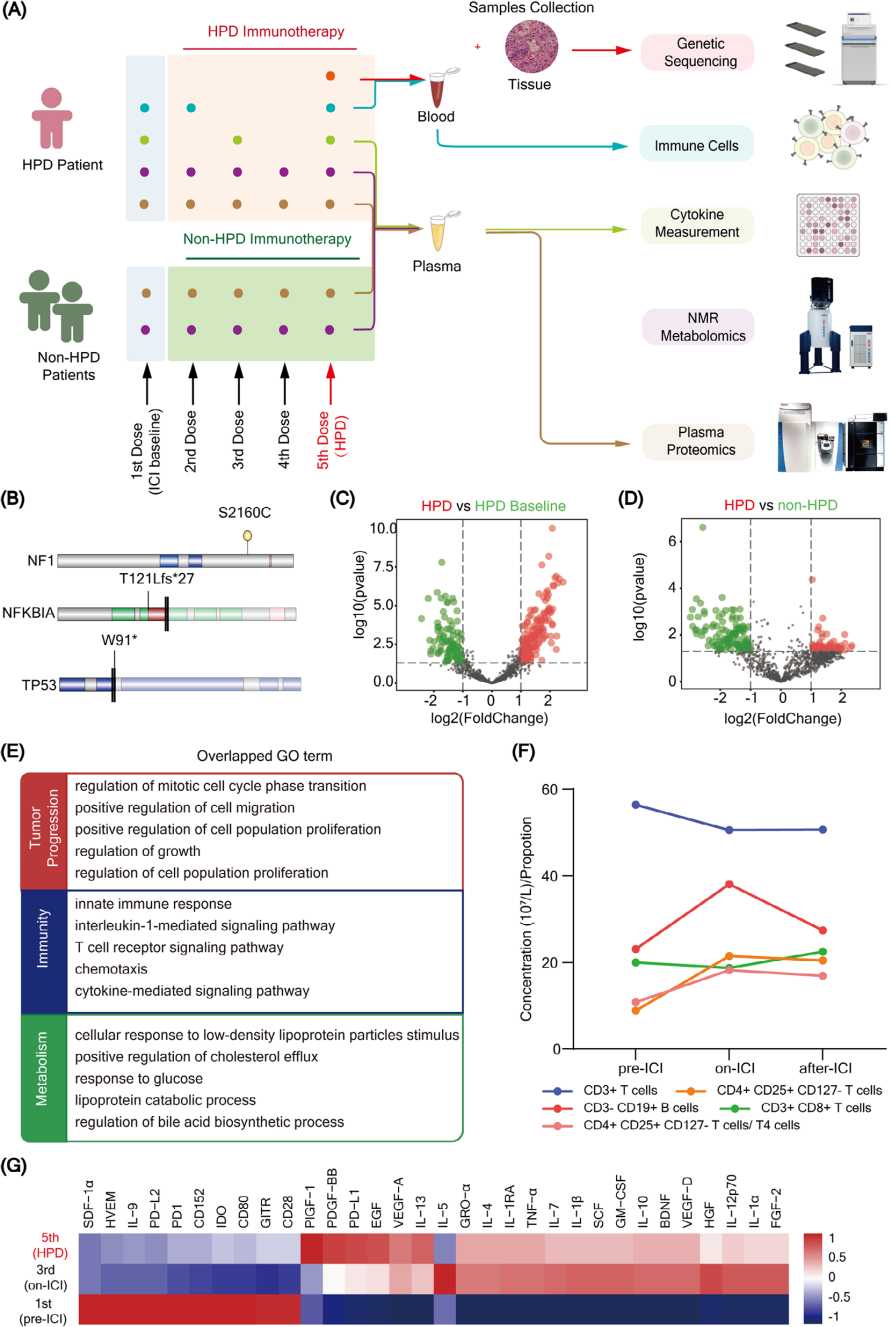
Molecular characteristics of patient with hyperprogression disease (HPD). (A) Workflow of multi‐omics research for enrolled patients. (B) Pathogenetic mutations detected in patients with HPD. (C, D) Volcano plot showing the relationship between each protein's fold change (FC) and the log10 of the *p*‐value from the moderate t‐test comparing each protein value between indicated groups. Proteins with FC  >  2 and moderate t‐test two‐sided *p*  <  .05 are shown in red. (E) Gene Ontology enrichment in patients with HPD. (F) Circulating immune cells significantly changed after immune checkpoint inhibitor (ICI) treatment in HPD. (G) Circulating cytokines alteration after ICI treatment in HPD.

To characterize the mechanism underlying HPD, a protein‐protein interaction network was constructed based on the mutated proteins (Figure S2A and Table S3). We further performed gene set enrichment analysis based on differential plasma proteins (Figure 1C, D and Tables S4 and S5). Three core functional classes, tumour progression, Th2 cell differentiation and metabolism, were identified (Figure 1E and Figure S2B). Activated tumour progression was expected,^4^ but the immune and metabolic disorders in HPD remain unclear. Dynamic changes of circulating immune cells in HPD showed an obvious increase in CD19+ B cells, and inhibitory CD4+CD25+ T cells after immunotherapy, but decreases in total T cells and CD8+ T cells (Figure 1F). Total T cells and CD8+ T cells tended to increase during ICI therapy but decreased after ICI therapy in patients without HPD (Figures S2C and S2D). Cytokine analysis revealed elevated levels of pro‐proliferative cytokines and Th2 cytokines, while T‐cell‐activating cytokines obviously decreased (Figure 1G and Table S6). These data suggest a suppressed immune profile for HPD, which is consistent with previously reported immune tumour microenvironment alterations of HPD.^5^

Plasma metabolomics was performed to explore metabolic dysregulation in HPD (Figure 2A). Low‐density lipoprotein (LDL) was significantly upregulated in HPD (Figure S3A–D). Orthogonal partial least squares‐discriminant analysis (OPLS‐DA) showed that plasma lipid metabolites can effectively differentiate HPD (Figure 2B,C). Many lipids were significantly dysregulated in HPD (Figure 2D,E). Partial correlation analysis with covariate adjustment for time was also performed (Figure 2F). Integrated with these results, we identified 15 key metabolites. Notably, these metabolites were predominantly concentrated in the LDL‐6 lipoprotein (Figure 2G), which was reported to be associated with cancer.^6^ LDL‐6 is a subtype of low‐density lipoprotein with the smallest particle size and highest density (Figure S3E). The proportion of LDL‐6 increased from 28.9% preimmunotherapy to 40.2% post‐HPD, while the average LDL diameter significantly decreased (Figure 2H, I and Table S7). LDL‐6 levels increased rapidly in HPD during ICIs therapy but remained stable or declined in patients without HPD (Figure 2J and Figure S3F–I). In addition, LDL‐6 triglyceride (L6TG) increased throughout the whole process, especially after the early immunotherapy, and even beyond the upper limit (Figure 2K,L). However, because only one patient was included, additional samples are needed in the future to validate the predictive value of LDL‐6 subfractions for HPD. Other than lipid metabolites, OPLS‐DA of nonlipid metabolites clearly distinguish samples from HPD (Figure S4A). To screen vital nonlipid metabolites, we integrated the results of OPLS‐DA (Figure S4A,B), differential metabolites (Figure S4C,D) and partial correlation analysis (Figure S4E). N, N‐dimethyglycine (DMG) and histidine stood out (Figure S4F). Patients with HPD exhibited a deceased DMG (Figure S4G) and histidine level (Figure S4H). Further analysis revealed that the histidine began to decrease below the lower limit after two weeks of immunotherapy (Figure S4I). Histidine and DMG dysregulation might be another metabolic characteristic in HPD, but further studies with larger sample sizes are needed. Altogether, our findings revealed that metabolic dysregulation serves as an important characteristic of HPD, which provided a new insight into HPD mechanisms.

**FIGURE 2:**
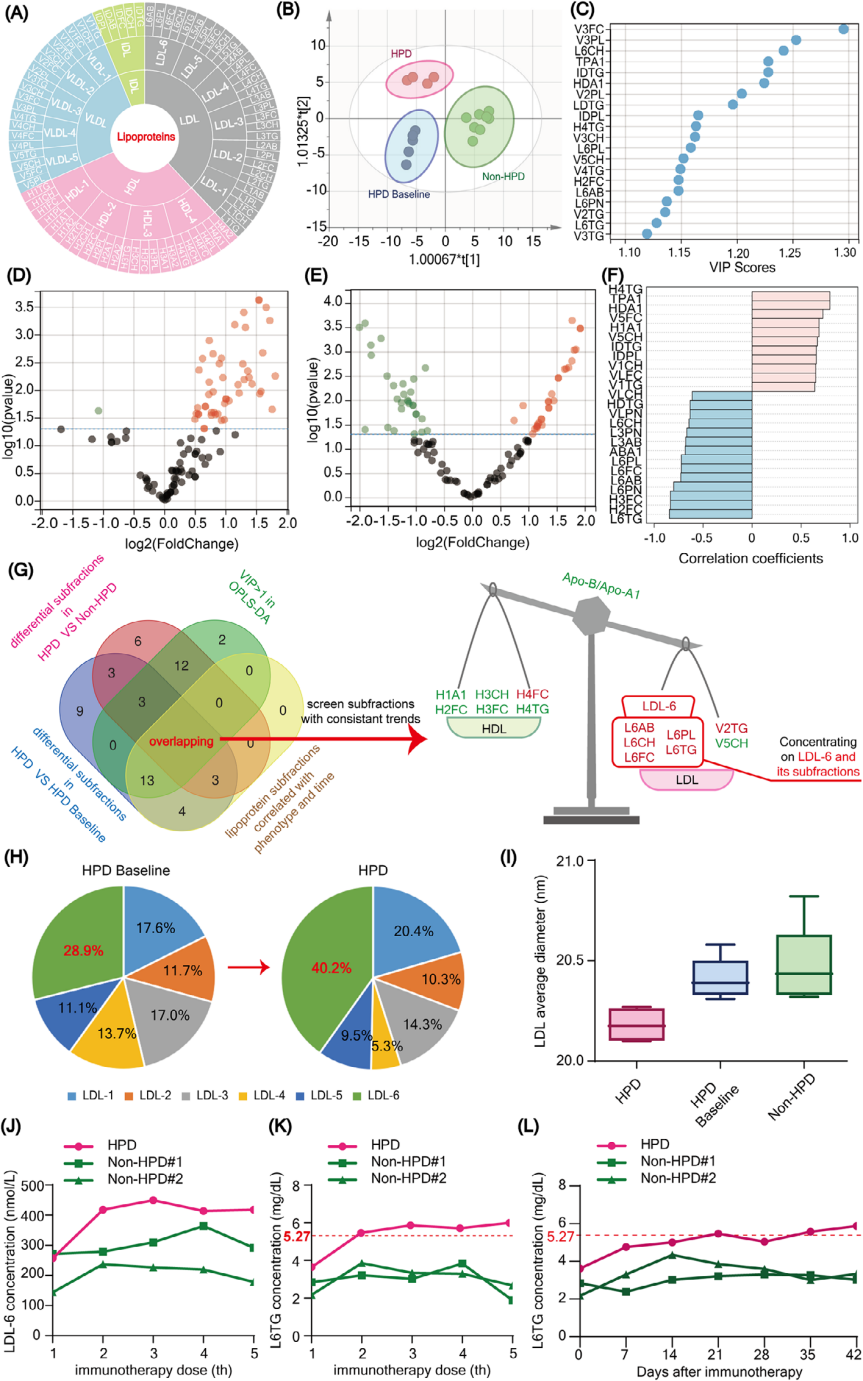
Lipoprotein subfractions of low‐density lipoprotein (LDL)‐6 in serum samples are increased in patients with hyperprogression disease (HPD). (A) Sub‐classification of lipoproteins detected by nuclear magnetic resonance (NMR). (B) Orthogonal partial least squares‐discriminant analysis (OPLS‐DA) analysis on lipoprotein subfractions. (C) Lipid metabolites ranked by variable importance in projection (VIP) scores in OPLS‐DA. (D, E) Volcano plot showing the relationship between each lipid metabolite's fold change (FC) and the log10 of the p‐value from the moderate t‐test comparing each protein value between indicated groups. Proteins with FC  >  2 and moderate t‐test two‐sided *p*  <  .05 are shown in orange. (F) Top 25 phenotype‐correlated lipids. (G) Overlapping lipoprotein subfractions showed a high‐density lipoprotein (HDL)‐LDL shift and obviously concentrated on the LDL‐6 subgroup. (H) Changes in LDL components, especially LDL‐6 proportion, before and after immunotherapy. (I) Composition change led to significantly smaller LDL particles in HPD. (J) Dynamic alteration of LDL‐6 particle numbers detected in HPD and Non‐HPD patients during the process of whole immune checkpoint inhibitor (ICI) treatment. (K, L) LDL‐6 subfractions L6TG dynamic changes during the process of whole ICI treatment and the early changes within 1.5 months after ICI treatment.

Metabolic biomarkers are preferable for dynamic monitoring of HPD because of their liability to be influenced by metabolic diseases.^7^, ^8^ Thus, we aimed to discover a protein biomarker to identify HPD. We first screened 65 differential proteins based on plasma proteomics (Figure 3A). We subsequently constructed an interacted network of these differential proteins with HPD mutated proteins (Figure 3B), analyzed the correlations of these differential proteins with differential plasma metabolites (Figure 3C and Table S8) and evaluated the prognostic value of these proteins (Figure 3D and Figure S5). After these screenings, SAA1 was the only candidate (Figure 3E). SAA1 is expressed mainly in malignant cells with single‐cell analysis (Figure 3F). Furthermore, the plasma SAA1 trend to increase during HPD throughout the whole process, and even started during the early period of ICIs treatment (Figure 3G,H). Due to the limitation of sample size, further studies with more samples are needed in the future to confirm these findings. A validation cohort with 10 patients with HPD and 21 patients without HPD was used to confirm the effectiveness of SAA1. The baseline characteristics between HPD and non‐HPD groups exhibited no differences (Table S9). SAA1 is expressed in tumour cells and highly expressed in HPD in various cancers (Figure 4A,B). Excitedly, 83.3% of patients with high SAA1 expression ultimately developed HPD without the influence of infection or metabolic disease^9^, ^10^ (Figure 4C,D). Therefore, these data suggest that SAA1 is a promising biomarker for the prediction of HPD prediction in pan cancers.

**FIGURE 3:**
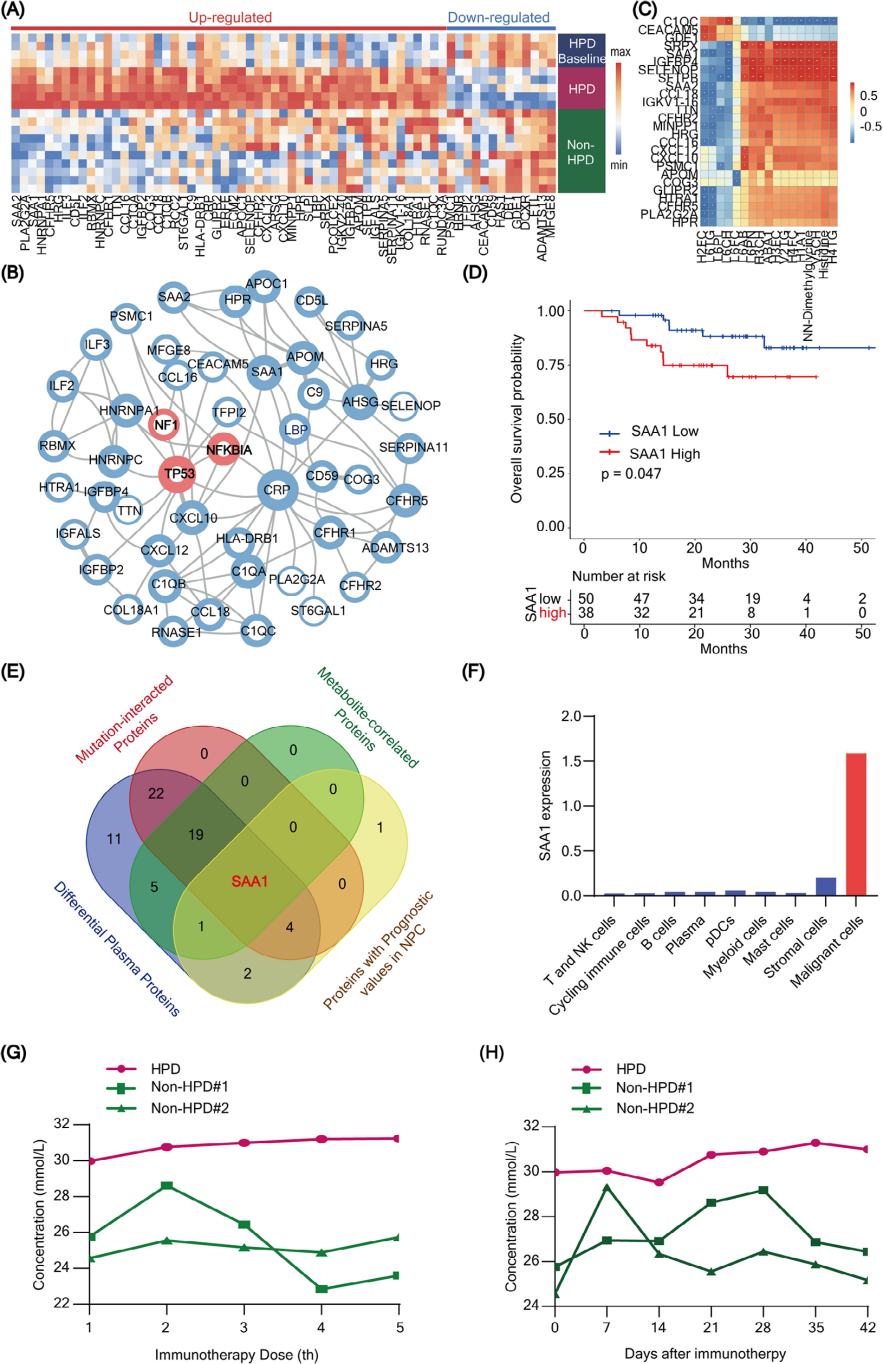
Screening potential predictive biomarker for hyperprogression disease (HPD). (A) Hot plot showing the differential plasma proteins between indicated groups. (B) Interacted network of potential mutations and differential plasma proteins in HPD. (C) Correlations among differential plasma proteins and differential plasma metabolites in HPD. (D) Overall survival (OS) of patients with high/low SAA1 expression, the *p*‐value was calculated using a log‐rank test. (E) Venn plot of overlapping candidate plasma proteins. (F) SAA1 distribution in diverse cell types is illustrated by single‐cell analysis. (G, H) Dynamic alterations of serum SAA1 during the process of whole immune checkpoint inhibitor (ICI) treatment and the early changes within 1.5 months after ICI treatment.

**FIGURE 4:**
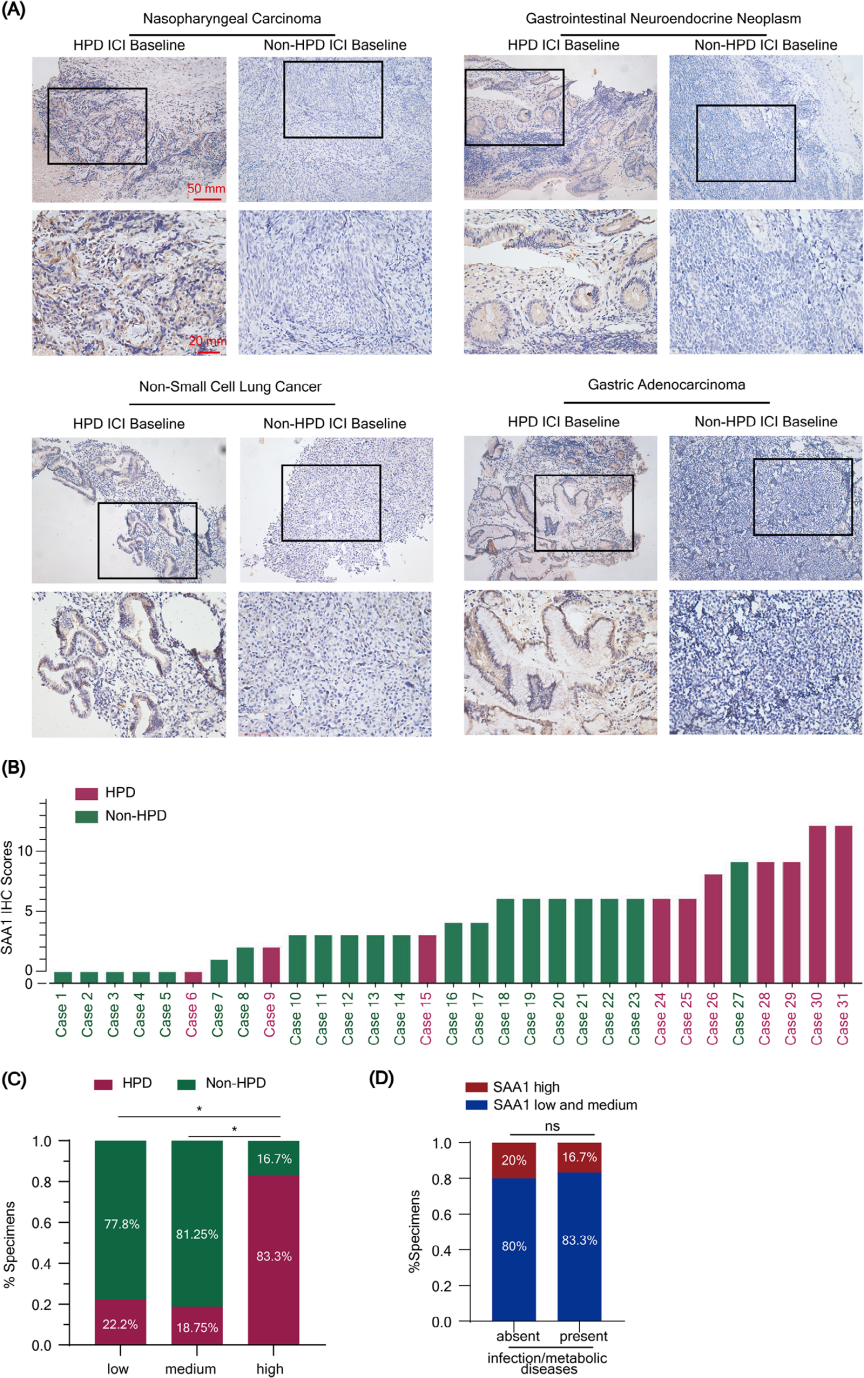
SAA1 could be a potential biomarker for hyperprogression prediction in pan cancers. (A) Representative images of SAA1 immunohistochemical (IHC) staining in hyperprogression disease (HPD) and non‐HPD cancer specimens in pan‐cancers (scale bar, 50 and 20 mm). (B) Bar plot showing the IHC scores of all enrolled patients, HPD are showing in violet red and Non‐HPD are showing in dark green. (C) Correlation between HPD and the expression level of SAA1. *p*‐Value was determined by Fisher's exact test. (D) Correlation between infection and metabolic disease and the expression level of SAA1. *p*‐Value was determined by Fisher's exact test. *, *p* < .05; ns, no significant.

In summary, our study provides a novel biomarker for HPD prediction, suggesting that easy immunohistochemical staining of SAA1 to predict HPD effectively.

## AUTHOR CONTRIBUTIONS

Jian Guan and Laiyu Liu conceptualized the study; Xiaoqing Wang, Longshan Zhang and Liwei Liao designed and performed most of the experiments; Nan Li and Tingxi Tang performed and supervised the proteomics analysis; Jianda Sun, Zhenhua Zhou and Jihong Huang carried out and supervised metabolite analysis; Yang Liu and Yingqiao Wang carried out and supervised the single cell analysis and survival analysis; Zekai Chen, Hanbin Zhang, Yunming Tian, Xiuting Zheng, Yi Yuan and Linlin Xiao were engaged in sample processing and clinical follow‐up; Xiaoqing Wang and Liwei Liao wrote the first version of the manuscript, Longshan Zhang and Jian Guan revised the manuscript. All authors have reviewed and agreed to the published version of the manuscript.

## CONFLICT OF INTEREST STATEMENT

The authors declare no conflict of interest.

## ETHICS STATEMENT

The patients’ consents for nasopharyngeal carcinoma were obtained and the patients’ consents of validation cohort were waived following the ethics committee of Nanfang Hospital protocol review. The study was approved by the ethics committee of Nanfang Hospital, Southern Medical University (project number NFEC‐2022‐470).

## Supporting information

Table S1. Plasma Samples information.Supporting Information

Table S2. Genes mutations detected by targeted next‐generation sequencing.Supporting Information

Talble S3. Gene Ontology Analysis of protein‐protein interaction network based on mutated proteins. Table S4. Gene Ontology Analysis of differential plasma proteins between HPD and HPD Baseline.Supporting Information

Table S5. Gene Ontology Analysis of differential plasma proteins between HPD and non‐HPD. Supporting Information

Table S6. Circulating cytokines detected at 1st/3rd/5th immunotherapy.Supporting Information

Table S7. Plasma LDL subtypes in HPD baseline and HPD status.Supporting Information

Supporting Information

Talbe S8 Correlations of differential plasma proteins with differential plasma metabolites. Supporting Information

Table S9 Immunotherapy baseline of validated cohort.Supporting Information

Table S10 The total proteomics data of all samples.Supporting Information

Talbe S11 The total metabolomics data of all samples.Supporting Information

SuppMat. Materials and methods; supplementary figures.Supporting Information

## Data Availability

The total proteomics data of all samples were provided in Table S10. The total metabolomics data of all samples were provided in Table S11.
