# Supplementary material for: SAA1 and metabolomic signatures predict hyperprogression with immunotherapy in pan cancers
Source: Clin Transl Med. 2024 Mar 11;14(3):e1624. doi: 10.1002/ctm2.1624 (PMC10928447; doi:10.1002/ctm2.1624)
Supplement: Supplementary file 1 — Table S1. Plasma Samples information.Supporting Information [file CTM2-14-e1624-s001.docx]

**Table S1.** **Plasma Samples information**

| patients |  | samle collected time | sample name | sample type |
| --- | --- | --- | --- | --- |
| HPD | Pre-ICIs |  | X566 | plasma |
|  |  |  | X618 | plasma |
|  |  |  | X667 | plasma |
|  |  |  | X672 | plasma |
|  | ICIs-1 | day0 | X675 | plasma |
|  |  | day1 | X683 | plasma |
|  |  | day3 | X689 | plasma |
|  |  | day7 | X692 | plasma |
|  |  | day14 | X702 | plasma |
|  | ICIs-2 | day21 | X714 | plasma |
|  |  | day22 | X721 | plasma |
|  |  | day24 | X725 | plasma |
|  |  | day28 | X732 | plasma |
|  |  | day35 | X742 | plasma |
|  | ICIs-3 | day42 | X751 | plasma |
|  | ICIs-4 | day63 | X779 | plasma |
|  | ICIs-5 | day84 | X821 | plasma |
| non-HPD#1 | ICIs-1 | day0 | X499 | plasma |
|  |  | day1 | X505 | plasma |
|  |  | day3 | X512 | plasma |
|  |  | day7 | X518 | plasma |
|  |  | day14 | X533 | plasma |
|  | ICIs-2 | day21 | X545 | plasma |
|  |  | day22 | X552 | plasma |
|  |  | day24 | X561 | plasma |
|  |  | day28 | X570 | plasma |
|  |  | day35 | X582 | plasma |
|  | ICIs-3 | day42 | X598 | plasma |
|  | ICIs-4 | day63 | X664 | plasma |
|  | ICIs-5 | day84 | X958 | plasma |
| non-HPD#2 | ICIs-1 | day0 | X501 | plasma |
|  |  | day1 | X507 | plasma |
|  |  | day3 | X514 | plasma |
|  |  | day7 | X520 | plasma |
|  |  | day14 | X535 | plasma |
|  | ICIs-2 | day21 | X547 | plasma |
|  |  | day22 | X554 | plasma |
|  |  | day24 | X563 | plasma |
|  |  | day28 | X572 | plasma |
|  |  | day35 | X584 | plasma |
|  | ICIs-3 | day42 | X600 | plasma |
|  | ICIs-4 | day63 | X697 | plasma |
|  | ICIs-5 | day84 | X737 | plasma |

ICIs: immune checkpoint inhibitors
