# Supplementary material for: SAA1 and metabolomic signatures predict hyperprogression with immunotherapy in pan cancers
Source: Clin Transl Med. 2024 Mar 11;14(3):e1624. doi: 10.1002/ctm2.1624 (PMC10928447; doi:10.1002/ctm2.1624)
Supplement: Supplementary file 2 — Table S2. Genes mutations detected by targeted next‐generation sequencing.Supporting Information [file CTM2-14-e1624-s002.docx]

| **Table S2. Genes mutations detected by targeted next-generation sequencing** | | | |  |
| --- | --- | --- | --- | --- |
| Mutations | Nucleotide changes & Amino acid changes | Mutation rate in plasma | Mutation rate in tumor tissue |  |
|  |  |  |  |  |
|  |  |  |  |  |
| CD74 | c.694_695delTG | 51.40% | 48.21% |  |
|  | p.C232Rfs*38 |  |  |  |
| FGF3 | c.503_518del16 | 23.83% | 23.17% |  |
|  | p.K168Rfs*108 |  |  |  |
| MSH2 | c.1318C>T | 1.13% | 2.14% |  |
|  | p.L440F |  |  |  |
| NF1 | c.6478A>T | 35.25% | 34.28% |  |
|  | p.S2160C |  |  |  |
| NFKBIA | c.349_397del49 | 28.08% | 29.45% |  |
|  | p.T121Lfs*27 |  |  |  |
| TP53 | c.273G>A | 49.21% | 50.71% |  |
|  | p.W91* |  |  |  |
