# Supplementary material for: SAA1 and metabolomic signatures predict hyperprogression with immunotherapy in pan cancers
Source: Clin Transl Med. 2024 Mar 11;14(3):e1624. doi: 10.1002/ctm2.1624 (PMC10928447; doi:10.1002/ctm2.1624)
Supplement: Supplementary file 6 — Table S7. Plasma LDL subtypes in HPD baseline and HPD status.Supporting Information [file CTM2-14-e1624-s009.docx]

| **Table S6. Circulating cytokines detected at 1st/3rd/5th immunotherapy** | | | |
| --- | --- | --- | --- |
| Cytokines (pg/ml) | 1st (pre-ICI) | 3rd (on-ICI) | 5th (HPD) |
| PlGF-1 | ND | 3.78 | 26.79 |
| GRO-a | ND | 15.16 | 13.2 |
| IL-1a | 0.25 | 4.83 | 3.28 |
| BDNF | 11.03 | 116.16 | 96.61 |
| IL-1RA | 154.38 | 1469.41 | 1262.62 |
| IL-10 | 0.99 | 9.27 | 7.56 |
| IL-4 | 2.87 | 25.81 | 22.83 |
| VEGF-D | 4.55 | 32.58 | 27.49 |
| IL-1b | 11.23 | 74 | 58.77 |
| IL-7 | 5.58 | 35.36 | 28.66 |
| VEGF-A | 33.85 | 181.02 | 202.35 |
| IL-12p70 | 2.19 | 12.23 | 9.31 |
| PDGF-BB | 26.89 | 96.4 | 164.64 |
| FGF-2 | 16.89 | 64.01 | 48.73 |
| TNF-a | 15.52 | 53.77 | 48.04 |
| HGF | 353.43 | 1330.77 | 923.24 |
| IL-5 | 9.74 | 46.03 | 11.14 |
| PD-L1 | 3.58 | 8.64 | 12.06 |
| SCF | 6.83 | 20.78 | 17.45 |
| GM-CSF | 65.71 | 161.75 | 138.94 |
| EGF | 5.12 | 9.73 | 12.67 |
| IL-13 | 12.55 | 24.84 | 29.67 |
| IL-18 | 41.89 | 83.97 | 63.54 |
| IFN-g | 26.4 | 41.57 | 41.07 |
| IP-10 | 41.73 | 54.47 | 55.57 |
| IL-6 | 108.27 | 164.58 | 114.39 |
| IL-2 | 67.33 | 84.71 | 80.5 |
| IL-15 | 114.09 | 134.32 | 141.68 |
| TNF-b | 125.28 | 121.99 | 173.4 |
| MIP-1b | 28.51 | 36.33 | 29.58 |
| IL-8 | 23.33 | 23.11 | 28.9 |
| CD27 | 455.44 | 373.89 | 560.35 |
| IL-21 | 122.06 | 90.63 | 142.65 |
| IL-22 | 168.48 | 134.08 | 164.84 |
| LIF | 90.31 | 78.45 | 81.52 |
| IL-27 | 622.09 | 442.4 | 654.34 |
| CD137 (4-1BB) | 77.76 | 54.31 | 79.27 |
| IL-17A | 80.71 | 67.76 | 65.26 |
| Eotaxin | 13.36 | 11.35 | 10.21 |
| TIM-3 | 1823.36 | 1104.57 | 1817.15 |
| RANTES | 82.88 | 71.54 | 59.62 |
| MIP-1a | 27.98 | 19.85 | 24.02 |
| IFN-a | 9.58 | 4.91 | 9.95 |
| BTLA | 1148.63 | 735.94 | 913.81 |
| MCP-1 | 40.29 | 34 | 23.41 |
| LAG-3 | 272.11 | 135.52 | 188.12 |
| IL-23 | 334.67 | 162.35 | 209.1 |
| NGF-b | 20.37 | 11.4 | 10.18 |
| IDO | 183.08 | 70.88 | 103.1 |
| SDF-1a | 524.06 | 236.12 | 236.12 |
| GITR | 183.76 | 56 | 99.57 |
| PD1 | 120.9 | 39.06 | 56.28 |
| CD80 | 846.18 | 245.86 | 417.38 |
| IL-9 | 80.01 | 25.33 | 32.37 |
| CD28 | 2055.82 | 476.66 | 1005.53 |
| CD152 | 228.99 | 57.34 | 97.36 |
| PD-L2 | 1733.84 | 440.96 | 585.24 |
| HVEM | 276.11 | 35.93 | 57.2 |
| PlGF-1 | ND | 3.78 | 26.79 |

ND: Not Detected.
