# Supplementary material for: SAA1 and metabolomic signatures predict hyperprogression with immunotherapy in pan cancers
Source: Clin Transl Med. 2024 Mar 11;14(3):e1624. doi: 10.1002/ctm2.1624 (PMC10928447; doi:10.1002/ctm2.1624)
Supplement: Supplementary file 7 — Supporting Information [file CTM2-14-e1624-s006.docx]

**Table S7. Plasma LDL subtypes in HPD baseline and HPD status**

| sample | HPD Baseline (nmol/L) | HPD (nmol/L) |
| --- | --- | --- |
| LDL-1 | 155.96 | 212.61 |
| LDL-2 | 103.93 | 107.58 |
| LDL-3 | 150.49 | 148.58 |
| LDL-4 | 121.11 | 54.96 |
| LDL-5 | 98.48 | 98.83 |
| LDL-6 | 256.43 | 417.7 |
