# Supplementary material for: SAA1 and metabolomic signatures predict hyperprogression with immunotherapy in pan cancers
Source: Clin Transl Med. 2024 Mar 11;14(3):e1624. doi: 10.1002/ctm2.1624 (PMC10928447; doi:10.1002/ctm2.1624)
Supplement: Supplementary file 9 — Table S9 Immunotherapy baseline of validated cohort.Supporting Information [file CTM2-14-e1624-s005.docx]

**Table S9.** **Immunotherapy baseline of validated cohort**

|  |  | HPD  (n = 10) | Non-HPD  (n = 21) | *P* |
| --- | --- | --- | --- | --- |
| Age | medium±SD | 47.9±14.33 | 56.43±11.32 | 0.082 |
| sex | male | 8(80%) | 18(85.7%) | 0.686 |
|  | famale | 2(20%) | 3(14.3%) |  |
| BMI | medium±SD | 21.07±2.38 | 22.92±3.76 | 0.148 |
| Smoking | Smoker | 5(50%) | 7(33.3%) | 0.373 |
|  | Non-smoker | 5(50%) | 14(66.7%) |  |
| Family history of cancer | Yes | 2(20%) | 3(14.3%) | 0.686 |
|  | No | 8(80%) | 18(85.7%) |  |
| History of infection and metabolic disorders | Yes | 1(10%) | 5(23.8%) | 0.363 |
|  | No | 9(90%) | 16(76.2%) |  |
| Tumor |  |  |  |  |
| T | 1 | 1(10%) | 2(9.5%) | 0.292 |
|  | 2 | 4(40%) | 2(9.5%) |  |
|  | 3 | 1(10%) | 5(23.8%) |  |
|  | 4 | 4(40%) | 10(47.6%) |  |
|  | x | 0 | 2(9.5%) |  |
| N | 0 | 0 | 1(4.8%) | 0.452 |
|  | 1 | 3(30%) | 2(9.5%) |  |
|  | 2 | 4(40%) | 6(28.6%) |  |
|  | 3 | 3(30%) | 9(42.8%) |  |
|  | x | 0 | 2(9.5%) |  |
| M | 0 | 5(50%) | 14(66.7%) | 0.373 |
|  | 1 | 5(50%) | 7(33.3%) |  |
| Stage | II | 1(10%) | 3(14.3%) | 0.702 |
|  | III | 2(20%) | 2(9.5%) |  |
|  | IV | 7(70%) | 16(76.2%) |  |
| Cancer type | NPC | 1(10%) | 2(9.5%) | 0.494 |
|  | NSCLC | 3(30%) | 9(42.8%) |  |
|  | GC | 5(50%) | 10(47.6%) |  |
|  | Others | 1(10%) | 0 |  |

HPD, Hyperprogression disease; BMI, Body Mass Index; NPC, nasopharyngeal carcinoma; NSCLC, non-small cell lung cancer; GC, gastric adenocarcinoma.
