# Supplementary material for: SAA1 and metabolomic signatures predict hyperprogression with immunotherapy in pan cancers
Source: Clin Transl Med. 2024 Mar 11;14(3):e1624. doi: 10.1002/ctm2.1624 (PMC10928447; doi:10.1002/ctm2.1624)
Supplement: Supplementary file 12 — SuppMat. Materials and methods; supplementary figures.Supporting Information [file CTM2-14-e1624-s010.docx]

**SUPPLEMENTARY INFORMATION**

**MATERIALS AND METHODS**

**1. Patients and samples**

3 patients with recurrent/metastasis nasopharyngeal carcinoma from prospective clinical trial (NCT05682703) were recruited in 2019 at Nanfang hospital, Guangzhou, China. 31 Patients with obtained biopsy tumor tissues in the baseline of immunotherapy who received immunotherapy during the period of 2018 till 2022 were enrolled as validation cohort. The baseline characteristics of this validation cohort were listed in supplementary Table S1. Venous blood samples were collected from all patients using EDTA Tubes, and then centrifuged at 1000 G for 10 min for plasma collection. The plasma samples were frozen at - 80 °C. Plasma Samples information was listed in supplementary Table S2. Tumor tissue samples were collected and immersed into 10% formalin and send for testing immediately.

**2. Plasma Proteomics Analysis**

Collected plasma samples were send to ProteinT (Tianjin) Biotech Co., Ltd for Proteomics Analysis. Briefly, 40 μL collected plasma was treated to remove the highly abundant proteins using PT plasma kit purchased from ProteinT (Tianjin) Biotech Co. Ltd. Plasma Proteomic analyses based on Nano-LC-MS/MS and performed on an Orbitrap Q Exactive HF mass spectrometer (Thermo Fisher Scientific) accompanied with a Thermo Scientific UltiMate 3000 UHPLC system.

**3. Metabolomics performed by NMR Spectroscopy**

Plasmas for lipoprotein subclasses detection and non-lipid metabolites were detected by Bruker 600 MHz Avance III NMR spectrometer. The commercial Bruker IVDr Lipoprotein Subclass Analysis (B.I.-LISA) method was adopted.^9^ Absolute concentrations were qualified by ERETIC method (Electronic Reference To access In vivo Concentrations).

**4. Immunohistochemistry (IHC)**

In brief, Paraffin embedded specimens were sliced into 2.5 μm sections. The sections were baked at 65°C for 2 hours and deparaffinized with xylenes and rehydrated. Next, sections were immersed in Tris-EDTA buffer and heated for antigen retrieval, and then incubated in 3% superoxol to block endogenous peroxidase for 15 min. The samples were then incubated with anti-SAA1 polyclonal antibody diluted at 1:100 (Proteintech) at 4 °C overnight. After washing with PBS, the sections were incubated in anti-rabbit secondary antibody at room temperature for 1 h. Next, diaminobenzidine (DAB) was used to reveal the color of antibody staining. Finally, the slides were mounted and observed under an optical microscope (Leica, Germany).

IHC staining was performed on 31 paraffin-embedded pan-cancer tissue sections using SAA1 antibodies. SAA1 staining was evaluated based on the proportion of positively stained tumor cells (0 for no positive cells, 1 for < 10% positive cells, 2 for 11%-50%, 3 for 51%-80%, and 4 for > 80%) and the staining intensity (0 for no staining, 1 for weak staining, 2 for moderate staining, and 3 for strong staining). The staining index (SI) calculated as the proportion of positive cells multiplied by the staining intensity, ranging from 0 to 12. The expression of SAA1 were divided into three grades as according to SI scores: 0-2, low expression; 3-6, medium expression; 8-12, high expression. Details of the IHC method were provided in the supplementary methods and materials.

**5. Target next generation sequencing**

Target next generation sequencing and variants analysis were performed at a commercial laboratory (Genecast Biotechnology Co., Ltd, Wuxi, China). A target NGS DNA panel of 543 cancer-related genes was used to detected genetic mutations, copy number variation (CNV), [microsatellite](https://cn.bing.com/dict/search?q=microsatellite&FORM=BDVSP6&cc=cn) [instability](https://cn.bing.com/dict/search?q=instability&FORM=BDVSP6&cc=cn) and tumor mutation burden (TMB).

**6. Cytokines assay**

Serums for circulating cytokines were tested in a commercial laboratory (Genecast Biotechnology Co., Ltd, Wuxi, China). Cytometric Bead immune Array was utilized for detection of 58 peripheral cytokines. Cytokines with concentration change more than 2 folds were considered as differential cytokines.

**7. Circulating immune cells analyses**

Circulating immune cells were analyzed during whole treatment process. Fresh peripheral blood was collected and tested for CD3+ cells, CD3+CD4+ cells, CD3+CD8+ cells, CD3-CD19+ cells, CD4+CD45RA cells, CD4+CD45RO cells, CD8+CD28+ cells, CD8+CD28- cells, CD4+CD25+CD127- cells and CD25 cells in clinical laboratory of Nanfang Hospital, Guangzhou.

**8. Public datasets collected**

For single-cell RNA sequencing, 38 NPC patients and 10 non-malignant individuals in this study were enrolled from three centres/laboratories (PMID: 33531485; 32901110; 33750785), from whom 48 biopsy and 10 peripheral blood samples were collected.

**9. Single-cell RNA-seq data processing**

Doublets are artefactual libraries generated from two cells arising due to errors in droplet encapsulation of cells, and thus commonly affect the quality of single-cell sequencing data. The R package “DoubletFinder” (version 2.0.3, <https://github.com/chris-mcginnis-ucsf/DoubletFinder>) was applied to predict doublets in our data. Basically, a doublet is defined as a single-cell library representing more than one cell, and a closer examination of some known markers would suggest that the offending cluster consists of doublets of more than one cell type, while no cell type is known to strongly express both markers at the same time. We removed doublets in each sample individually, with an expected doublet rate of 0.05 and default parameters used otherwise. The remaining cells survived from the filtering criteria were single cells. Then the gene expression matrices for all remaining cells were combined and converted to a Seurat object using the R package Seurat (version 4.0.0, <https://satijalab.org/seurat>). Next, any cells were removed for which had either less than 1001 UMIs, or expression of less than 501 genes, or over 25% UMIs linked to mitochondrial genes. From the remaining cells, gene expression matrices were generated with log normalization and linear regression using the NormalizeData and ScaleData function of the Seurat package.

Because the samples were processing independently and high-dimensional variables are common in single-cell sequencing data, which might introduce potential batch effect. Therefore, we used harmony algorithm to remove batch effect. Next, we used RunUMAP function implemented in Seurat to reduce dimensionality. Cell clusters were identified using the FindClusters function in Seurat, with a K parameter of 30 and default parameters used otherwise. We annotated the clusters as different major cell types based on their average gene expression of well-known markers, including T (*CD3D*), NK (*KLRF1*), myeloid (*AIF1*), malignant (*EPCAM*), B (*MS4A1*), plasma (*MZB1*), mast (*TPSB2*), pDC (*LILRA4*), endothelial (*VWF*), fibroblasts (*COL1A1*), and cycling immune (HMGB2) cells.

**10. Bulk RNA sequencing and data analysis**

We collected 88 NPC RNA-sequencing data with prognostic information from the public dataset GSE102349. We assessed the associations of genes and survival of NPC. Receiver operating characteristic (ROC) was used to determine the optimal cut-off value of gene expression for patient stratification. Kaplan-Meier analysis was conducted to reveal the prognostic ability of the expression levels of genes in 88 NPC samples with prognostic information, and a two-sided log-rank test was performed to compare the survival between high and low expression levels of genes.

**11.Statistical analysis**

Statistical analyses were performed and visualized by GraphPad Prism 9 software. All results considered p < 0.05 significant. Functions of mutated proteins were predicted by PROVEAN (Protein Variation Effect Analyzer) software. Interacted proteins were predicted by STRING (https://cn.string-db.org/). KEGG Orthology-Based Annotation System (KOBAS) was used to perform KEGG pathway enrichment analyses. Statistics normalization, Partial correlation and Multivariate Empirical Bayes Analysis of Variance (MEBA) analysis were analyzed by MetaboAnalyst statistical analysis tool web service (https://www.metaboanalyst.ca/) (5). Orthogonal partial least squares discriminant analysis (OPLS-DA) was performed using SIMCA 14.1 software (MKS Data Analytics Solutions, Umeå, Sweden). Metabolites with variable influence on projection (VIP) score > 1 were selected. Student’s t test was performed and visualized into volcano plot by Sangerbox 3.0 (http://vip.sangerbox.com/home.html). Common metabolites were overlapped and plotted by Bioinformatics & Evolutionary Genomics (http://bioinformatics.psb.ugent.be/webtools/Venn/).

**Supplementary Figures and Figure Legends**

**
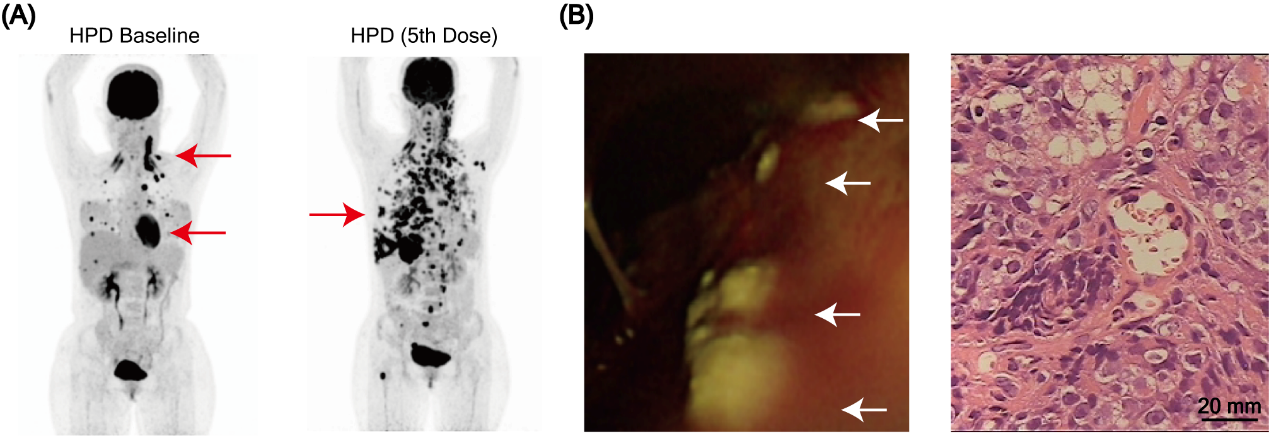
**

Figure S1. Imaging and pathologic data of HPD patient. (A) PET-CT image before (at baseline) and after (diagnosed HPD) immunotherapy. (B) Image of thoracoscopy after (diagnosed HPD) immunotherapy and representative image of HE staining in tumor tissue from thoracoscopic biopsy.


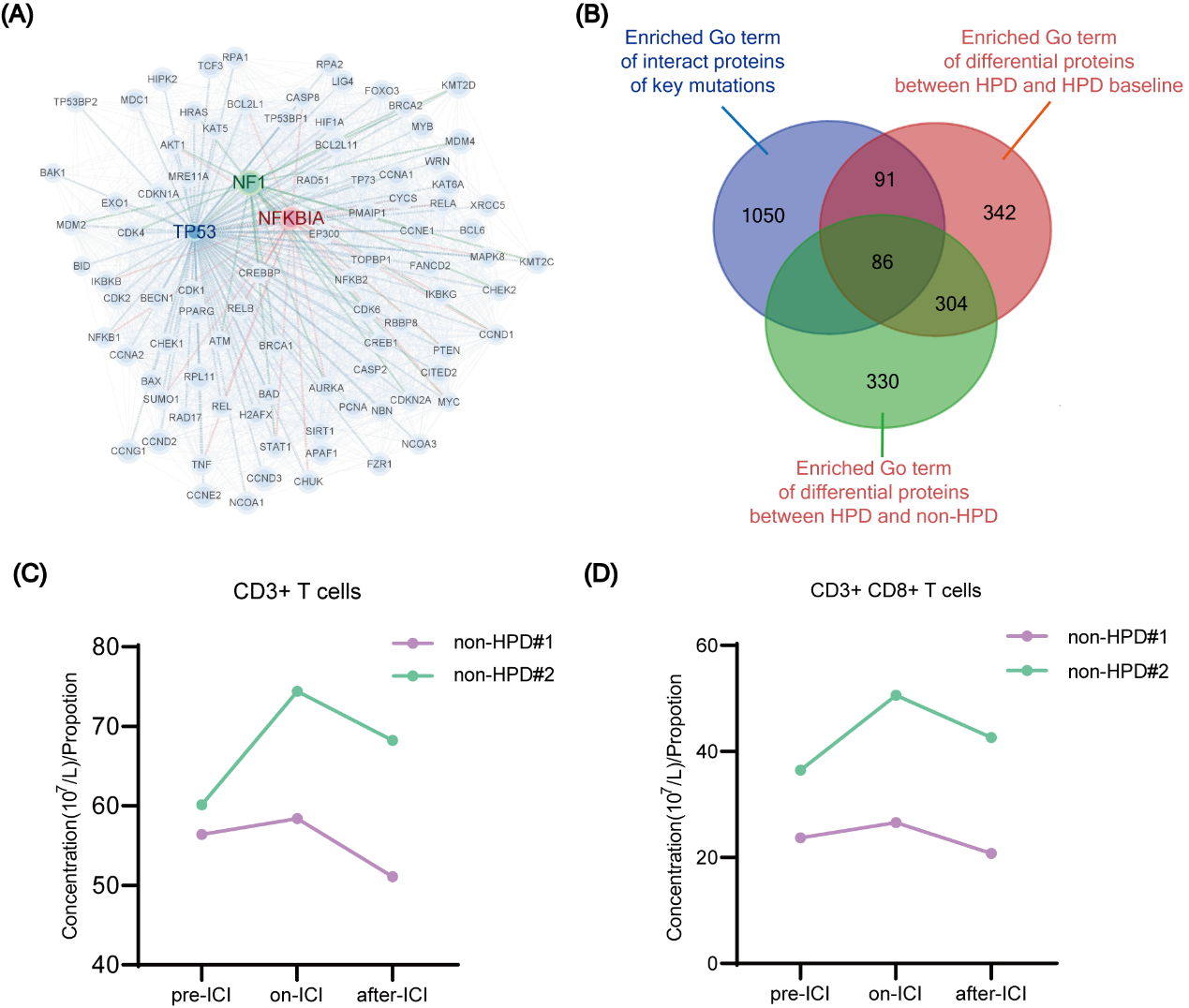


Figure S2. Protein-protein interaction network of mutate proteins and GO Term in HPD. (A) protein-protein interaction network for NF1, TP53 and NFKBIA by STRING. (B) Venn plot of overlapping KEGG pathway between differential plasma proteins and interact proteins of key mutations. (C) Circulating CD3+ T cells in patients without HPD. (D) Circulating CD3+ CD8+ T cells in patients without HPD.

**
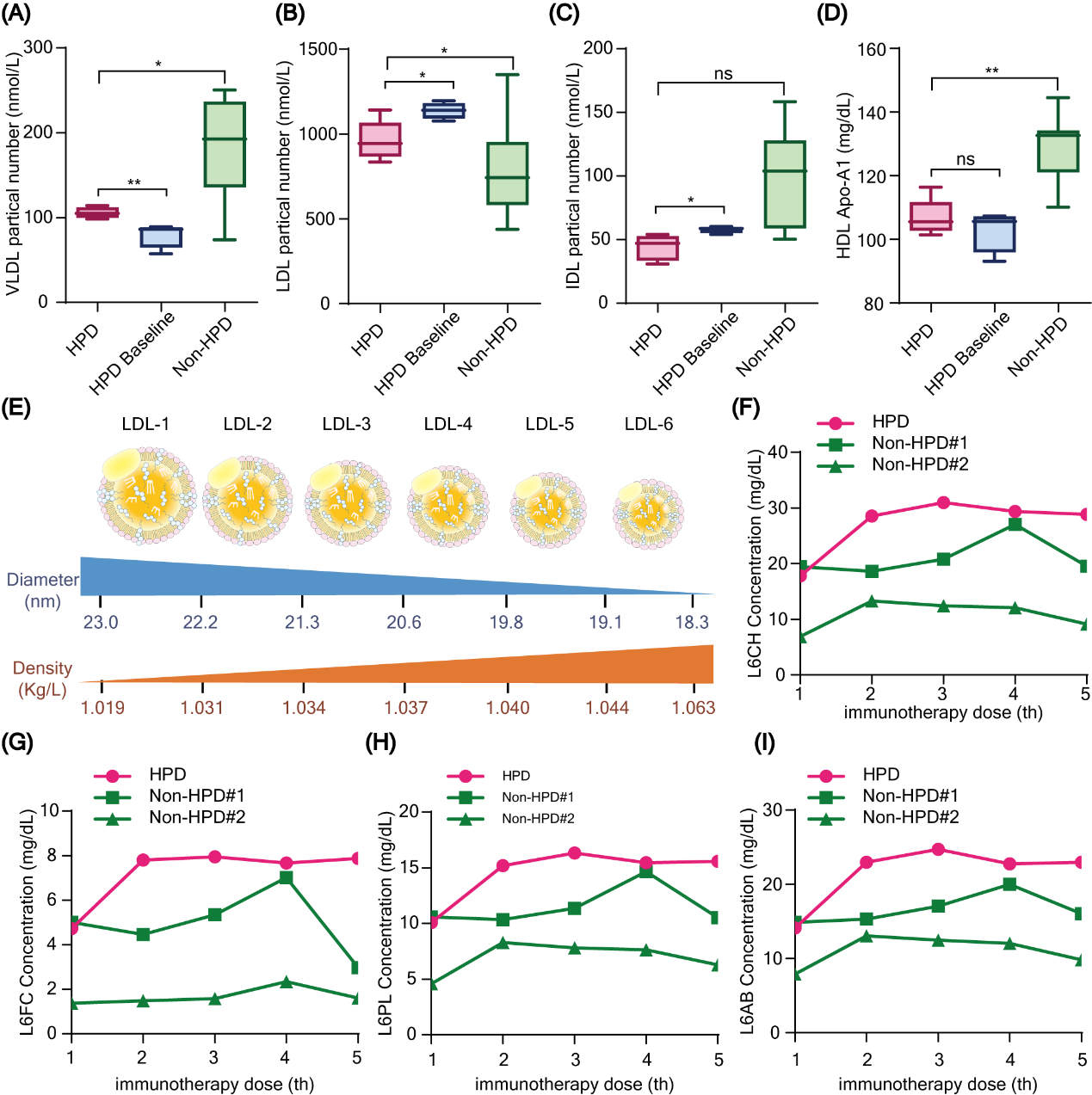
**

Figure S3. LDL dysregulation in HPD. (A-D) Comparison of lipoprotein particle concentrations of VLDL (A), LDL (B), IDL (C) and HDL (D). (E) Presentation of LDL subtypes, LDL-6 is the smallest and densest one. (F-I) Dynamic alterations of dLDL-6 subfractions of L6CH (F), L6CF (G), L6PL (H) and L6AB (I) during the process of whole ICI treatment.

**
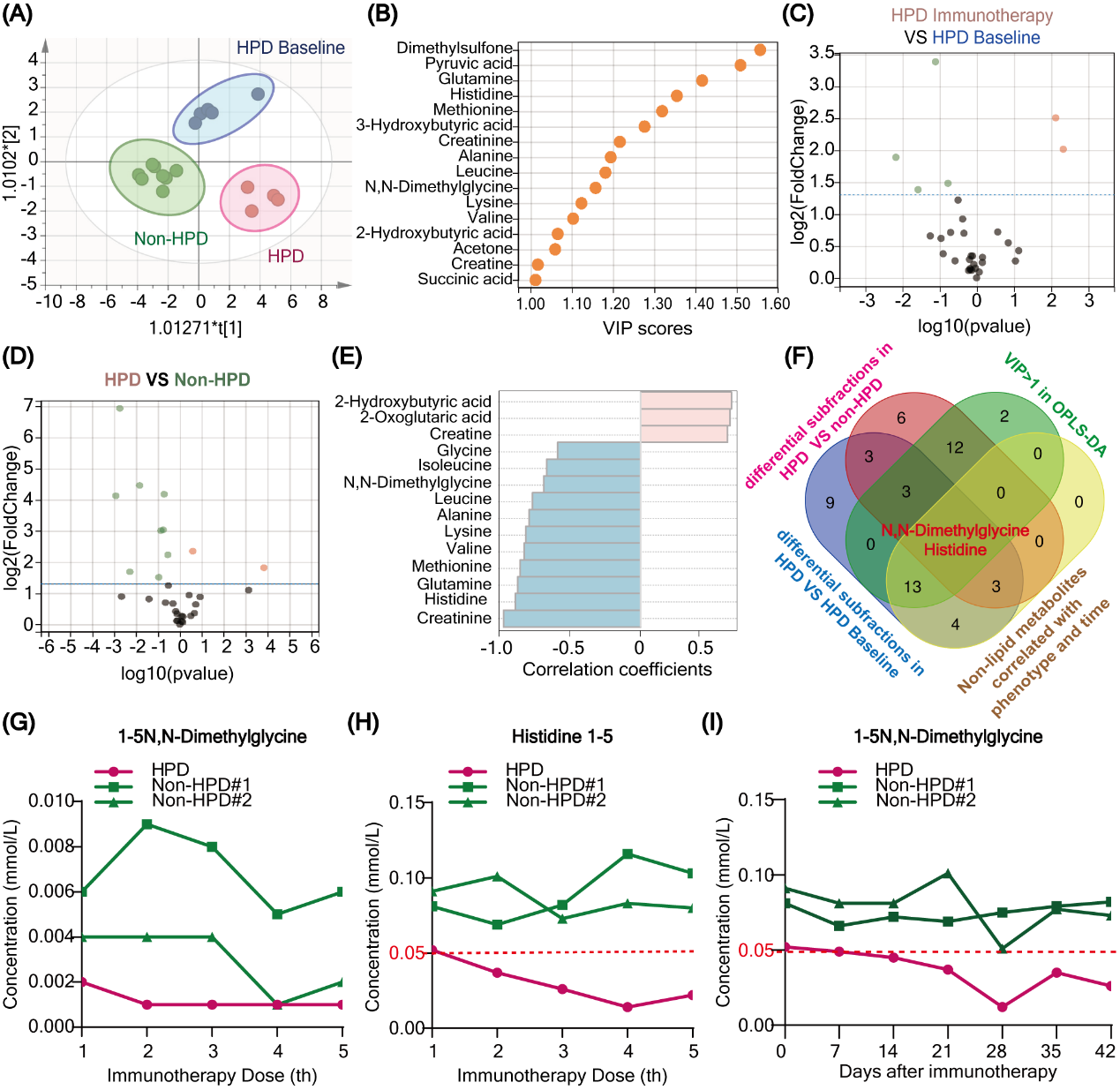
**

Figure S4. Non-lipid metabolic dysregulation in HPD. (A) OPLS-DA analysis on non-lipid metabolites. (B) Lipid metabolites ranked by VIP scores in OPLS-DA. (C-D) Volcano plot showing the relationship between each non-lipid metabolites’ fold change (FC) and the log10 of the p value from the moderate t-test comparing each protein value between indicated groups. Proteins with FC > 2 and moderate t-test two-sided p < 0.05 are shown in orange. (E) Phenotype-correlated non-lipids metabolites. (F) Venn plot of overlapping non-lipid metabolites. (G) Dynamic alteration of 1.5 N, N-dimethyglycine in HPD and Non-HPD patients during the process of whole ICI treatment. (H-I) Histidine dynamic changes during the process of whole ICI treatment and the early changes with 1.5 months after ICI treatment.

**
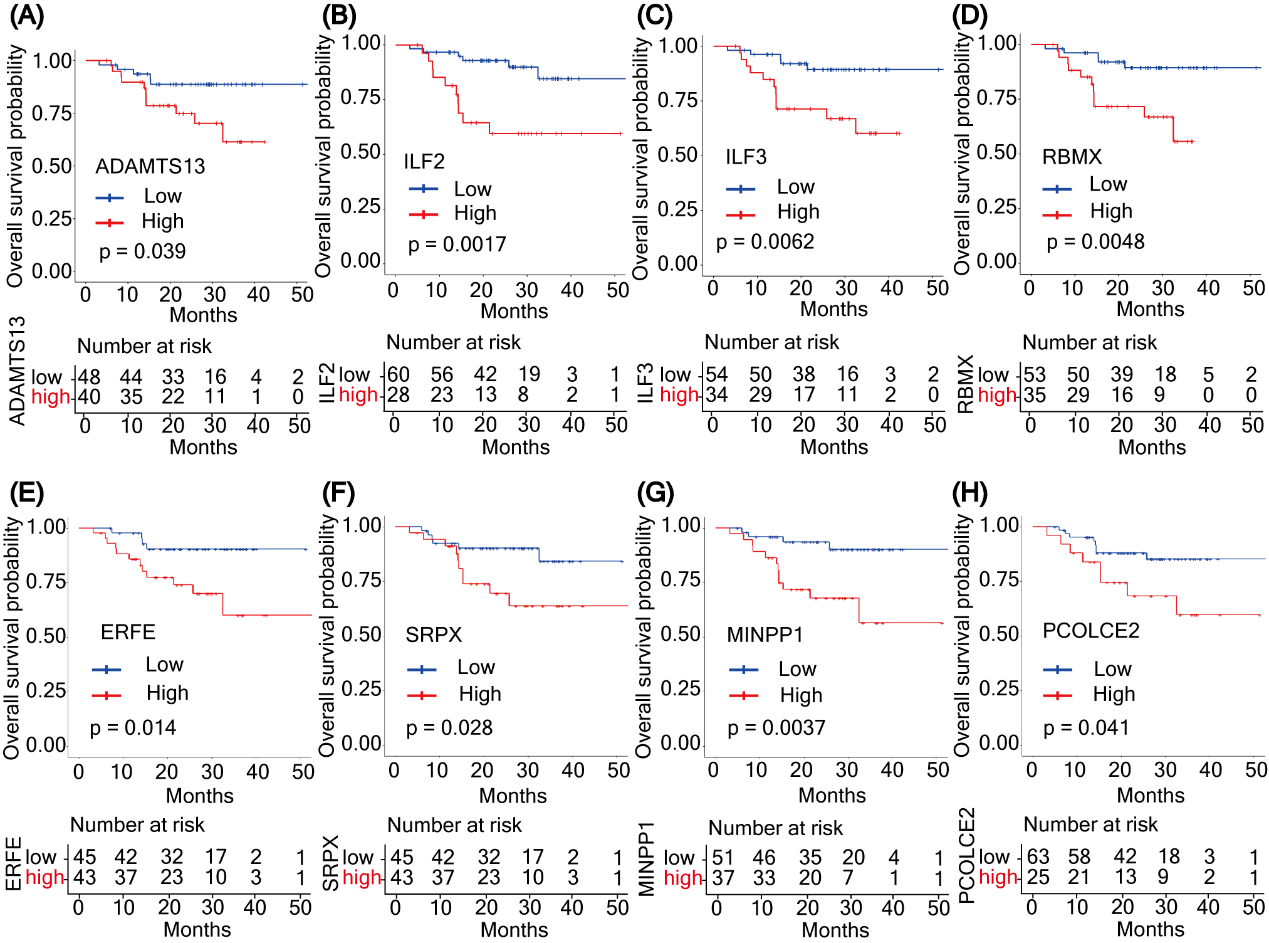
**

Figure S5. Kaplan Meier plot of indicated proteins in nasopharyngeal carcinoma. (A) OS of patients with high/low ADAMTS13 expression. (B) OS of patients with high/low ILF2 expression. (C) OS of patients with high/low ILF3 expression. (D) OS of patients with high/low RBMX expression. (E) OS of patients with high/low ERFE expression. (F) OS of patients with high/low SRPX expression. (G) OS of patients with high/low MINPP1 expression. (H) OS of patients with high/low PCOLCE2 expression. All the *p* value was calculated using a log-rank test.
